# Use of En Bloc Kidney Allografts for Pediatric Kidney‐Alone and Multiorgan Transplant Recipients in the United States

**DOI:** 10.1111/petr.70330

**Published:** 2026-05-13

**Authors:** Rachel M. Engen, James D. Perkins, Lyndsay A. Harshman, Clare Lindner, Juhi Kumar, Dechu Puliyanda, Sharon M. Bartosh, Jodi M. Smith

**Affiliations:** ^1^ University of Wisconsin Madison Madison Wisconsin USA; ^2^ Clinical and Bio‐Analytics Transplant Laboratory University of Washington Seattle Washington USA; ^3^ Organ Transplant Center, University of Iowa Iowa City Iowa USA; ^4^ University of Michigan Ann Arbor Michigan USA; ^5^ University of Pittsburgh Pittsburgh Pennsylvania USA; ^6^ Cedars Sinai Guerin Children's Los Angeles California USA; ^7^ University of Washington Seattle Washington USA

**Keywords:** en bloc transplantation, organ allocation, outcomes, pediatric kidney transplantation

## Abstract

**Background:**

En bloc kidney transplantation is controversial, but recent adult studies increasingly show outcomes that are similar to or superior to those of high‐quality single kidney transplants. The use of en bloc allografts in children in the modern allocation era has not been reported.

**Methods:**

We compared SRTR data on en bloc kidney transplants in pediatric recipients 2000–2014 and 2015–2024 to better understand trends in utilization and outcomes in pediatric recipients.

**Results:**

There were 176 en bloc transplants in pediatric recipients, 138 in the 2000–2014 era, and 38 in the 2015–2024 era. 43.7% of en bloc transplants were part of a multiorgan transplant. Between 2013 and 2024, there was a decreasing use of en bloc allografts overall and specifically for kidney‐alone recipients. En bloc kidney‐alone transplants had decreased graft survival compared with single kidney transplants in the first year posttransplant, but similar outcomes thereafter. En bloc kidney‐multiorgan transplants had superior graft survival after 1 year posttransplant compared with single kidney‐alone transplants.

**Conclusion:**

Utilization of en bloc kidneys has declined in pediatric recipients but can have good outcomes in a select population. More information is needed to understand whether and how best to use en bloc kidney transplantation for adolescents with reduced access to transplant.

## Graphical abstract

Despite policy changes in the United States designed to increase the use of en bloc kidneys, utilization in pediatric recipients has declined sharply over time. In the current era, en bloc kidneys are used primarily in pediatric multiorgan recipients. However, in a select population, they can have good long‐term outcomes.

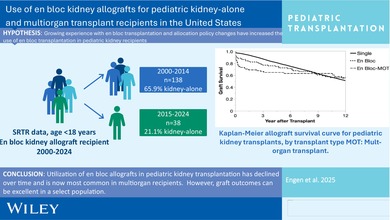

**Abbreviations**
CAKUT: congenital anomaly of the kidney and urinary tract
cPRA: calculated panel reactive antibody
DCD: donation after circulatory death
GN/FSGS: glomerulonephritis or focal segmental glomerulosclerosis
HLA: human leukocyte antigen
HR: hazard ratio
IQR: intrerquartile range
KAS: Kidney Allocation System
KDPI: Kidney Donor Profile Index
MOT: multiorgan transplant
OPTN: Organ Procurement and Transplantation Network
RMST: restricted mean survival time
SRTR: Scientific Registry of Transplant Recipients
UNOS: United Network for Organ Sharing

## 1 Background

En bloc kidney transplantation entails the transplantation of two kidneys from a single donor into a single recipient using the donor aorta and vena cava for vascular anastomosis. The technique was designed to allow the use of small pediatric donor organs while minimizing the risk of vascular complications and low nephron mass [1]. Among adult recipients, historical literature suggests that en bloc kidney transplants may have higher rates of vascular [2, 3, 4] and urologic [5] complications, leading to lower 1‐year allograft survival [3]. However, several recent studies have reported long‐term graft function [3, 6, 7, 8] and survival [8, 9, 10, 11] of en bloc donor kidneys in adult recipients which are similar to or exceeds high‐quality single kidney transplants. A single‐center analysis by Sureshkumar et al. showed similar graft survival among 72 en bloc kidney transplants compared to 75 living donor transplants at up to 27 years of follow‐up [8]. A 2024 study by White et al. using Scientific Registry of Transplant Recipient (SRTR) data reported that en bloc kidneys from donors ≥ 8 kg had similar 1‐year graft survival to single kidney allografts with a KDPI < 20%, while kidneys from donors < 8 kg had lower 1‐year graft survival but similar 10‐year graft survival compared with KDPI < 20% donor kidneys [9].

Among United States pediatric kidney transplant programs, the use of pediatric donor kidneys has historically been limited, both by concerns about graft thrombosis and by the structure of allocation policy [12]. Most data on outcomes of en bloc kidney transplantation into children come from single centers reporting excellent outcomes among a small number of patients [13, 14, 15]. An analysis of SRTR data 2000–2013 by Winnicki et al. found that pediatric recipients of an en bloc transplant had a shorter wait time to transplantation, similar hazard for graft failure, and better eGFR than pediatric recipients of a standard criteria donor kidney, though graft survival was numerically lower at 1 year [16].

Since the analysis by Winnicki et al., en bloc kidney allocation in the United States has undergone numerous changes, including the 2014 introduction of KDPI and the Kidney Allocation System (KAS) [17], the 2017 policy requiring kidneys from donors < 18 kg to be offered en bloc [18], and the 2019 policy update offering all kidneys from donors < 20 kg according to Sequence A (similar to single kidneys with KDPI < 20%) [19]. The primary objective of this study was to characterize whether growing experience with en bloc transplantation, coupled with allocation policy changes, has changed the use of en bloc transplantation in pediatric kidney recipients.

## 2 Methods

This is a retrospective cohort study using data from SRTR between January 1, 2000, and 31 December, 2024. Individuals included in the study received an en bloc kidney transplant before the age of 18 years, either as a kidney‐alone transplant or in combination with another organ. The cohort was divided into two groups, pre‐ January 1, 2015 and post‐ January 1, 2015, to account for changes in kidney allocation policy introduced in December 2014. Primary outcomes of interest included the trend in en bloc organ utilization over time as well as demographic and clinical characteristics of pediatric en bloc allograft recipients.

Secondary outcomes of interest for kidney‐alone recipients included graft loss prior to 1 year posttransplant, especially due to primary nonfunction/graft thrombosis, and long‐term graft survival. For this analysis, pediatric recipients of an en bloc kidney‐alone transplant were compared with pediatric recipients of a pediatric single deceased donor kidney between January 1, 2000, and 31 December, 2024. Potential confounders of interest included recipient age at transplant, receipt of a multiorgan transplant, and transplant center experience.

The optimal time split for separating early and late hazard effects between transplant groups was identified using a cutpoint analysis based on interaction modeling, which evaluates where the difference in hazards between periods is most pronounced. This analysis indicated that the strongest distinction occurred at 1 year. Restricted mean survival time (RMST) analysis was then performed before and after this 1‐year split, providing an absolute measure of survival time differences in months between groups. Finally, Cox proportional hazards models were fitted separately for the early (≤ 1 year) and late (> 1 year) periods, quantifying the relative hazards of graft failure for each group compared with those for the reference groups of single kidney‐alone deceased donor transplants.

All results with a *p*‐value < 0.05 were considered statistically significant. Statistical analyses were performed using JMP‐Pro Version 17.0.0 (SAS Institute Inc., Cary, NC, USA) and Python Version 3.13. Python analyses utilized relevant statistical and survival time analysis libraries, including lifelines and *statsmodels*. Descriptive statistics report counts and percentages for categorical data and medians and interquartile ranges for continuous data. Descriptive variables were compared using chi‐squared tests of independence for categorical variables and the Wilcoxon rank‐sum test for continuous variables. Graft survival at 1 year posttransplant for kidney‐alone recipients was compared using chi‐squared tests of independence. Multivariable survival analysis was not performed due to the small cohort size.

The data reported here have been supplied by the United Network for Organ Sharing (UNOS) as the contractor for the Organ Procurement and Transplantation Network (OPTN). The interpretation and reporting of these data are the responsibility of the authors and in no way should be seen as an official policy of or interpretation by the OPTN or the US government. This research was deemed exempt by the University of Washington Institutional Review Board.

## 3 Results

### 3.1 En Bloc Kidney Transplants in Pediatric Recipients 2000–2024

We identified 176 en bloc kidney transplants in pediatric recipients between January 1, 2000, and 31 December, 2024. Ninety‐seven of these transplants (55%) were kidney‐alone, while the remaining 79 (45%) were part of a multiorgan transplant (Table 1). Median age at transplant was 8.5 years (IQR 3–13.8 years). Thirty‐five percentage of en bloc transplants were preemptive, the median recipient cPRA was 0%, and 10% were second transplants. The median age of the en bloc donor was 1 year (IQR 1–3 years), and the median weight was 12.2 kg (IQR 9.5–15 kg) (Table 2). Median donor–recipient height ratio was 0.76 (IQR 0.6–0.96), and median donor–recipient weight ratio was 0.53 (IQR 0.28–0.84).

**TABLE 1:** Demographic and clinical characteristics of pediatric recipients of an en bloc kidney transplant, 2000–2024, overall and by transplant allocation era.

	2000–2024 (*N* = 176)		2000–2014 (*N* = 138)		2015–2024 (*N* = 38)		*p*
	*N*/med	%/(IQR)	*N*/med	%/(IQR)	*N*/med	%/(IQR)	
Age, years	8.5	(3–13.8)	10	(3–14)	4.5	(2–9.5)	0.02
Male	94	53.4%	73	52.9%	21	55.3%	0.56
Height, cm	117	(85.2–146.9)	128.3	(87.6–149)	99.2	(82–127.6)	0.01
Weight, kg	23.8	(13.6–42)	28.8	(14–45.9)	18	(13.3–29.1)	0.049
Blood type							0.99
A	56	31.8%	43	31.2%	13	34.2%	
AB	5	2.8%	4	2.9%	1	2.6%	
B	20	11.4%	16	11.6%	4	10.5%	
O	95	54%	75	54.4%	20	52.6%	
Race/ethnicity							0.95
Asian	6	3.4%	4	2.9%	2	5.3%	
Black	33	18.8%	26	18.8%	7	18.4%	
Hispanic	45	25.6%	35	25.4%	10	26.3%	
Other	7	4%	6	4.4%	1	2.6%	
White	85	48.3%	67	48.6%	18	47.4%	
Diagnosis							0.42
CAKUT	27	15.3%	22	15.9%	5	13.2%	
Genetic	28	15.9%	22	15.9%	6	15.8%	
GN/FSGS	23	13.1%	21	15.2%	2	5.3%	
Other	80	45.5%	59	42.8%	21	55.3%	
Re‐transplant	18	10.2%	14	10.1%	4	10.5%	
Dialysis							0.32
Preemptive	62	35.2%	45	32.6%	17	44.7%	
≤ 1 year	50	28.4%	42	30.4%	8	21.1%	
> 1 year– ≤ 3 years	45	25.6%	39	28.3%	7	18.4%	
> 3 years– ≤ 5 years	12	6.8%	8	5.8%	4	10.5%	
≥ 5 years	6	3.4%	4	2.9%	2	5.3%	
cPRA	0	(0–0)	0	(0–0)	0	(0–3)	0.56
Missing	25		20	14.5%	5	13.2%	
Transplant type							< 0.001
Kidney alone	99	56.3%	91	65.9%	8	21.1%	
Heart–Kidney	2	1.1%	0		2	5.3%	
Liver–Kidney	36	20.5%	22	15.9%	14	36.8%	
Liver–Kidney–Intestine	31	17.6%	21	15.2%	10	26.3%	
Liver–Kidney–Intestine–Pancreas	8	4.5%	10	26.3%	4	10.5%	

Abbreviations: CAKUT, congenital anomaly of the kidney and urinary tract; cPRA, calculated panel reactive antibody; GN/FSGS, glomerulonephritis/focal segmental glomerulosclerosis.

**TABLE 2:** Demographic and clinical characteristics of en bloc kidney donors for pediatric recipients, 2000–2024, overall and by transplant allocation era.

	2000–2024 (*N* = 176)		2000–2014 (*N* = 138)		2015–2024 (*N* = 38)		*p*
	*N*/med	%/(IQR)	*N*/med	%/(IQR)	*N*/med	%/(IQR)	
Age, years	1	(1–3)	1	(0–3)	2	(1–3)	0.25
Height, cm	86.4	(73.7–98)	86.2	(71.8–98.3)	89	(81.2–98.3)	0.31
Weight, kg	12.2	(9.5–15)	12	(9.3–15)	13.7	(11.6–15.8)	0.06
Donor–Recipient height ratio	0.76	(0.6–0.96)	0.74	(0.57–0.91)	0.89	(0.75–1.02)	0.002
Donor–Recipient weight ratio	0.53	(0.28–0.84)	0.5	(0.25–0.82)	0.73	(0.46–1.01)	0.007
Donor–Recipient BSA index							0.001
0 ≤ 0.65	90	51.1%	79	57.3%	11	29%	
> 0.65 ≤ 1	54	30.7%	27	26.8%	17	44.7%	
> 1 ≤ 1.5	25	14.2%	15	10.9%	10	26.3%	
> 1.5	7	4%	7	5.1%	0		
ABO incompatible	1	0.5%	1	0.7%	0		1
KDPI (%)	48	(39–50)	49	(43–58)	44	(39–50)	0.01
DCD donor	1	0.5%	1	0.7%	0		
HLA mismatch							0.6
0	4	2.3%	3	2.2%	1	2.6%	
1–2	7	4%	7	5%	0		
3	19	10.8%	14	10.1%	5	13.2%	
4	44	25%	35	25.4%	9	23.7%	
5	74	42%	59	42.8%	15	39.5%	
6	28	15.9%	20	14.5%	8	21.1%	

Abbreviations: DCD, deceased after cardiac death; HLA, human leukocyte antigen; KDPI, Kidney Donor Profile Index.

**FIGURE 1:**
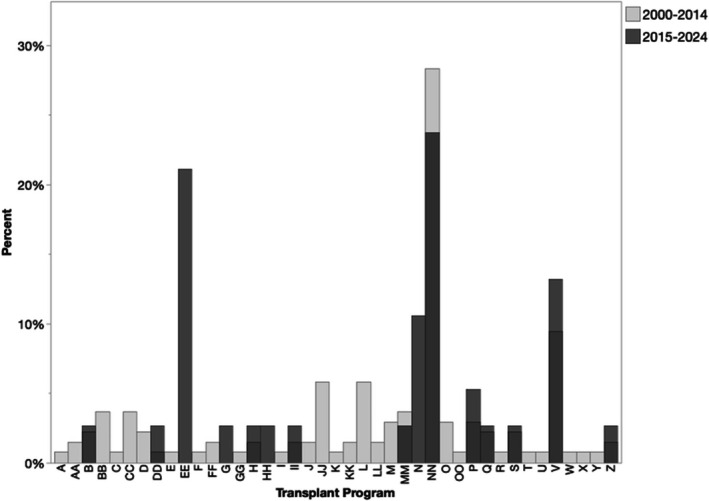
Percentage of en bloc kidney transplants into pediatric recipients performed by each transplant center, by era. Letter codes denote de‐identified transplant centers.

Geographic differences were found in the use of en bloc kidneys for pediatric recipients (Table 3). Region 3 performed 34.7% of all pediatric en bloc transplants, followed by Region 2 (14.2%) and Region 8 (10.2%). En bloc kidney usage was lowest in Regions 6, 4, and 1. Forty‐one transplant programs performed at least one en bloc kidney transplant in a pediatric recipient; four centers performed 69% of all pediatric en bloc transplants (Figure 1).

**TABLE 3:** Number of en bloc kidney transplants into pediatric recipients by region, overall and subdivided by allocation policy era. Percentage change in the average number of transplants per year in the 2015–2024 era compared with that in the 2000–2014 era is also shown.

Region	2000–2024 (*N* = 176)		2000–2014 (*N* = 138)		2015–2024 (*N* = 38)		% Change
	*N*	%	*N*	%	*N*	%	
1	5	2.8%	4	2.9%	1	2.6%	−62.5%
2	25	14.2%	23	16.7%	2	5.3%	−87%
3	61	34.7%	50	26.2%	11	29%	−67%
4	4	2.3%	4	2.9%	0		−100%
5	17	9.7%	16	11.6%	1	2.6%	−90.6%
6	1	0.6%	0	0	1	2.6%	+100%
7	12	6.8%	11	8%	1	2.6%	−86.4%
8	18	10.2%	13	9.4%	5	13.2%	−42.3%
9	7	4%	2	1.5%	5	13.2%	+275%
10	10	5.7%	7	5.1%	3	7.9%	−35.7%
11	16	9.1%	8	5.8%	8	21.1%	+50%

### 3.2 Trends in En Bloc Kidney Transplants Over Time

**FIGURE 2:**
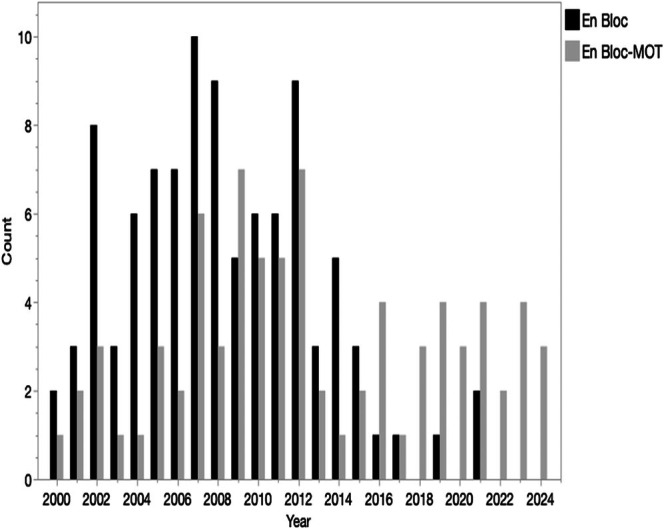
Number of en bloc kidney transplants into children performed each year 2000–2024, by transplant type (kidney‐alone versus multiorgan). MOT, multiorgan transplant.

En bloc kidney use in pediatric recipients remained uncommon throughout the study time, but there was a shift in the types of patients receiving them (Figure 2). From 2000 through 2005, there were an average of 6.7 pediatric en bloc transplants per year, with 73% of these being kidney‐alone transplants. From 2006 through 2012, the use of en bloc transplants rose to 12.4 per year, with 40% now being part of a multiorgan transplant. From 2013 through 2024, there were an average of 4 en bloc transplants per year, with 67.3% performed as part of a multiorgan transplant. No kidney‐alone en bloc transplants were reported between 2022 and 2024 (Table S1).

In the 2 years prior to the implementation of KAS in December 2014, there was an average of 5.5 en bloc transplants in pediatric recipients per year. In the 3 years after the implementation of KAS, 2015–2017, this declined to 2.5 per year. Following the 2017 “Improving Allocation of En Bloc Kidneys” policy change, pediatric use of en bloc kidney transplants dropped to 1.6 transplants per year, and in the 3 years after the 2019 “Improving Allocation of En Bloc Kidneys – 2” policy, usage rose to an average of 3.6 per year. For comparison, there were 2425 en bloc transplants performed in adult recipients during 2010–2014, representing approximately 161.7 transplants per year or 1.5% of all adult kidney transplants. Between 2015 and 2024, there were 1953 en bloc transplants into adult recipients, approximately 195.3 per year, but only representing 1.1% of all adult deceased donor transplants.

To better understand en bloc transplants under KAS, we compared the use of en bloc kidney transplants in pediatric recipients before and after January 1, 2015. Pediatric recipients of an en bloc kidney transplant in the post‐KAS era were significantly younger (median age pre‐KAS 10 years (IQR 3–14 years) vs. post‐KAS 4.5 years (IQR 2–9.5 years) *p* = 0.02) and smaller. They were also more likely to be listed for a multiorgan transplant (34.1% pre‐KAS vs. 78.9% post‐KAS, *p* < 0.001). There was no significant change in the characteristics of en bloc donors.

Geographic variation in en bloc transplantation shifted over time, though Region 3 remained the most common location (26.2% of en bloc transplants pre‐KAS and 29% post‐KAS). Pre‐KAS, Regions 2 and 5 were the second and third most common areas for pediatric en bloc transplant, while post‐KAS saw a shift toward Regions 11, 9, and 8. Of the 11 Organ Procurement and Transplant Network geographic regions, eight performed fewer en bloc transplants into children in the post‐KAS era, with the largest absolute declines in Regions 2, 3, 5, and 7.

At the program level, 39 out of 43 (90.1%) pediatric kidney transplant centers performed at least one pediatric en bloc transplant in the pre‐KAS era (Table S2). Post‐KAS, 28 out of those 39 centers performed zero pediatric en bloc transplants (71.8%). Nine centers continued performing pediatric en bloc transplants, but at a median 47.8% reduction in the proportion of pediatric transplants using an en bloc kidney. Six centers showed increased use of en bloc kidneys as a percentage of all pediatric kidney transplants; four of these had not used en bloc kidneys in the pre‐KAS era. Seven centers performed five or more pediatric en bloc kidney transplants in the pre‐KAS era, representing 60.1% of all pediatric en bloc transplants. In the post‐KAS era, four of these seven centers did not perform any pediatric en bloc transplants, while the other three centers all reported a reduction in use relative to single kidneys (median −58.5%, range −58.2% to −70.2%).

### 3.3 En Bloc Kidney Transplants in Multiorgan Recipients

Overall, 45% of en bloc pediatric kidney transplants were part of a multiorgan transplant. The most common pediatric multiorgan transplants using en bloc kidneys were liver–kidney (*n* = 36, 46%) and liver–kidney–intestine transplant (*n* = 31, 39%). En bloc kidney‐multiorgan recipients were younger than en bloc kidney‐alone recipients (4 years [IQR 2–8 years] vs. 13 years [IQR 7.5–16 years], *p* < 0.001) (Table S3). They also had a shorter duration of dialysis than en bloc kidney‐alone recipients: 57% of en bloc kidney‐multiorgan transplants were preemptive compared with 17.5% of en bloc kidney‐alone transplants (*p* < 0.001). En bloc kidney‐multiorgan donors had a slightly lower KDPI (46%, [IQR 40%–54%]) than en bloc kidney‐alone donors (49%, [IQR 43%–58%] *p* = 0.03), but there were no other significant differences in donor factors (Table S4).

### 3.4 Outcomes of En Bloc Kidney Transplants in Pediatric Recipients

**FIGURE 3:**
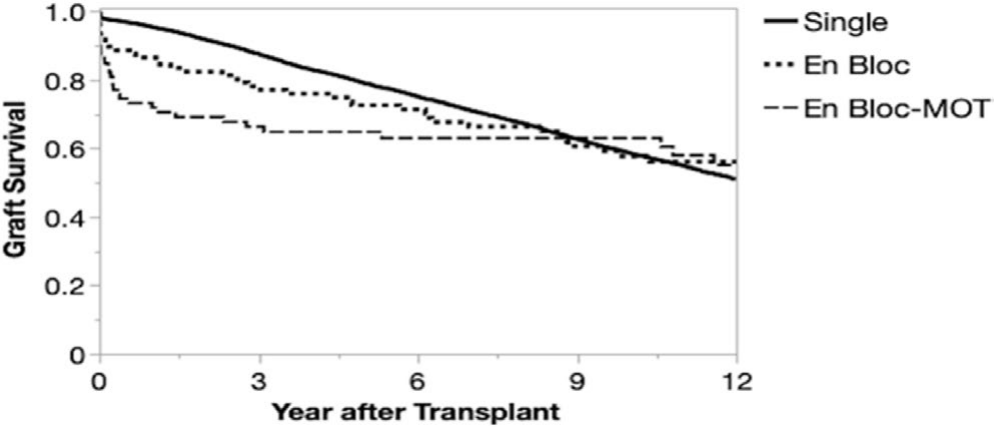
Kaplan–Meier allograft survival curve for pediatric kidney transplants performed 2000–2024 in the United States, by transplant type (single kidney vs. en bloc kidney‐alone vs. en bloc multiorgan). MOT, multiorgan transplant.

In a cohort comparing graft survival of en bloc kidney‐alone, en bloc kidney‐multiorgan, and single pediatric‐donor kidney‐alone transplants in pediatric recipients, single pediatric‐donor kidney‐alone transplants had superior survival at 1 year posttransplant (Table 4). This was driven primarily by lower 1‐year graft survival for en bloc transplants. In the first year posttransplant, en bloc kidney‐alone recipients had an average 0.9‐month lower graft survival than single pediatric‐donor kidney recipients (95% CI −1.6 to −0.2 months, *p* = 0.01), while en bloc kidney‐multiorgan recipients demonstrated 1.7 months lower graft survival (95% CI −2.7 to −0.9 months, *p* = 0.001). The primary cause of en bloc allograft failure was thrombosis (42.9%) (Table 5). After 1 year, there was no significant difference in graft survival between single kidney‐alone and en bloc kidney‐alone recipients (*p* = 0.47), while en bloc kidney‐multiorgan recipients had an average of 15.3 months longer kidney graft survival than single kidney‐alone recipients (95% CI 3.9–25.2 months, *p* = 0.01) (Figure 3).

**TABLE 4:** Kidney allograft survival of en bloc kidney‐alone and en bloc multiorgan in pediatric recipients, 2000–2024, compared with that in pediatric single kidney allograft recipients from pediatric donors only.

Graft survival	Single kidney			En bloc kidney‐alone			En bloc multiorgan		
Time posttransplant	*n*	%	*p*	*n*	%	*p*	*n*	%	*p*
1 year	2480	95.0%	Ref.	83	86.6%	< 0.001	56	71.9%	< 0.001
3 years	2001	86.4%	Ref.	74	78.2%	0.01	46	66.4%	< 0.001
6 years	1355	74.8%	Ref.	59	71.4%	0.23	33	63%	< 0.001
9 years	885	63.4%	Ref.	43	60.7%	0.32	27	63%	0.03
12 years	524	52.6%	Ref.	29	56.1%	0.75	19	55.2%	0.09

Abbreviation: Ref., Reference group.

**TABLE 5:** Kidney graft survival of en bloc kidney‐alone and en bloc kidney‐multiorgan transplants compared with that of single kidney‐alone recipients from pediatric donors, 2000–2024, in the first‐year posttransplant and after 1 year posttransplant.

	En bloc kidney‐alone			En bloc multiorgan		
		95% CI	*p*		95% CI	*p*
RSMT ≤ 1 year (months)	−0.9	(−1.6, −0.2)	0.01	−1.7	(−2.7, −0.9)	0.001
HR	2.75	(1.52–4.96)	< 0.001	5.31	(3.20–8.80)	< 0.001
RMST > 1 year (months)	3.6	(−6.8, 12.9)	0.47	15.3	(3.9, 25.2)	0.01
HR	0.79	(0.53–1.18)	0.25	0.47	(0.24–0.89)	0.02

Abbreviations: HR, cox proportional hazard ratio; RMST, restricted mean survival time.

There was no difference in graft survival at 6 years posttransplant between single pediatric‐donor kidney (74.8%) and en bloc kidney‐alone (71.4%) recipients (*p* = 0.23). All three transplant types had similar graft survival by 9 years posttransplant (single pediatric‐donor kidney 63.4%, en bloc kidney‐alone 60.7%, en bloc multiorgan 63%).

In an analysis limited to en bloc kidney‐alone transplants subdivided by era, recipients in the post‐KAS era had a significantly longer median waitlist time (268 days (IQR 157–1657 days)) than those in the pre‐KAS era (125 days (IQR 45–297 days), *p* = 0.014) (Table 6). There was no statistical difference in preemptive transplant. In the pre‐KAS era (*n* = 89), graft survival at 1 year was 85.4% with 42.9% of graft losses attributed to thrombosis and 14.3% attributed to primary nonfunction. There was one reported patient death within 1 year posttransplant. The number of en bloc kidney‐alone transplants in the post‐KAS era was very small (*n* = 8), but this select population reported excellent outcomes with no delayed graft function, graft loss within 1 year posttransplant, or patient death within 1 year posttransplant (Table 6).

**TABLE 6:** Outcomes of en bloc kidney‐alone transplants into pediatric recipients, 2000–2024, by transplant allocation era.

	2000–2014 (*n* = 89)		2015–2024 (*n* = 8)		*p*
	n/med	%/IQR	n/med	%/IQR	
Recipient age (years)	13	(7–16)	14	(9–17)	0.3
Dialysis duration (years)	0.9	(0.3–1.8)	1.1	(0.7–3.6)	0.29
cPRA	0	(0–0)	3	(0–78)	0.06
Wait list time, days	125	(45–297)	268	(157–1657)	0.01
Delayed graft function	10	11.2%	0		1
Preemptive transplant	16	18%	1	12.5%	1
Creatinine at 1 year (mg/dl)	0.7	(0.5–0.9)	0.8	(0.7–1.3)	0.55
Patient survival at 1 year	88	98.9%	8	100%	1
Graft survival at 1 year	76	85.4%	8	100%	0.59
Thrombosis	6	42.9%			
Primary nonfunction	2	14.3%			
Acute rejection	2	14.3%			
Other	2	14.3%			
BK virus	1	7.1%			

## 4 Discussion

In 2014, Winnicki et al. reported increased transplant access and good long‐term outcomes for pediatric recipients of an en bloc kidney transplant [16]; an editorial associated with that study suggested that these results “may help to increase the use of this still underutilized kidney transplant option in children… in an effort to get more children off dialysis” [20]. Since that time, the OPTN has made several changes designed to improve en bloc kidney utilization. A 2017 policy change mandated that kidneys from donors < 18 kg in body weight be allocated, en bloc, according to the same criteria as KDPI < 20% kidneys [18]. A 2019 policy update expanded the en bloc donor pool to donors < 20 kg, dictated that KDPI would not be reported for en bloc kidneys, and limited en bloc allocation to transplant centers that explicitly state a willingness to accept these kidneys [19]. Despite this, the use of en bloc kidneys in children in the United States has declined over the past 10 years, with en bloc kidney‐alone pediatric transplantation all but disappearing. The change in en bloc kidney utilization in pediatric recipients does not seem to correlate with any of these policy changes; rather, the notable drop‐off around 2013 could be explained by the decrease in pediatric composite visceral (aka multivisceral) transplants, which peaked in 2014–2015 and have been declining ever since [21]. Alternative explanations could relate to center‐specific changes in organ acceptance practices and surgical expertise.

An unexpected finding in this study was the relatively high use of en bloc kidneys in pediatric multiorgan recipients, especially as less than 70 pediatric multiorgan transplants are performed every year [22]. To our knowledge, this is the first comprehensive report of en bloc kidney use in pediatric multiorgan transplant. The majority of the transplants in our cohort included a liver, and this is likely related to the emphasis on donor–recipient size matching in liver transplantation, where large‐for‐size grafts are associated with graft compression, poor perfusion, and liver graft dysfunction [23]. A 2011 study by Harps et al. suggested a donor–recipient weight ratio of 3.4 as the cutoff for a good ICU outcome after pediatric liver transplantation, which is substantially above the median of 0.74 reported in our cohort [24]. Pediatric liver–kidney recipients are known to have decreased 1‐year graft and patient survival relative to kidney‐alone recipients, frequently related to early perioperative mortality, but have significantly higher eGFR, lower incidence of kidney graft rejection, and similar incidence of graft failure in the long‐term [25]. Similar improved long‐term outcomes were seen in our cohort, where en bloc kidney‐multiorgan recipients had a 55% lower hazard of graft failure relative to single kidney‐alone recipients. It has been hypothesized that this success could be due to an immunoprotective effect from the same‐donor liver [26, 27], though a kidney survival benefit in liver–kidney transplantation has not been reported in all studies [28].

En bloc kidneys often grow after transplant, with one study demonstrating that en bloc kidneys from infant donors more than doubled in the first 6 months posttransplant [29]. This may explain why adult studies have shown an improvement in eGFR in en bloc kidney transplant recipients over time [29, 30] and why studies of adult and pediatric en bloc kidney‐alone transplant recipients have similar long‐term outcomes to single kidney‐alone recipients, despite higher rates of early complications [8, 9, 16]. This finding was replicated in our cohort, where there was a substantially higher risk of graft failure in the first year posttransplant, but outcomes after 1 year were similar to those of single kidney recipients. However, our results were driven entirely by early graft failures in the pre‐2015 era. In the highly select and very small (*n* = 8) cohort of en bloc kidney‐alone transplants, outcomes at 1 year were excellent, with 100% graft survival.

Our study is the first to use national registry data to comprehensively describe the use of en bloc kidney transplantation in all pediatric recipients, allowing us to describe long‐term outcomes and avoid the impacts of center‐specific practices. However, our study is limited by incomplete data on the cause of kidney failure and a high level of missingness for cPRA and height at 1 year posttransplant (which limited our ability to assess eGFR). Registry data also cannot be used to assess surgical techniques, surgical complications, or management strategies, such as anticoagulation. Additionally, due to the small numbers of patients, we assessed outcomes for both primary and repeat transplant in a single cohort. While the number of repeat transplants was relatively small (< 10%), this may have introduced some confounding in the outcomes.

Our study shows that en bloc kidney transplantation remains uncommon in pediatrics, with many of the cases occurring in pediatric multiorgan recipients. Graft survival in the first year is significantly worse when compared to single kidney recipients but is improved thereafter. More recent en bloc kidney alone transplants are rare, but outcomes in this highly select population are excellent. This raises the question as to whether en bloc transplantation can be beneficial option for select patients, particularly older adolescents who are highly sensitized or have other barriers to transplant access. However, this should be performed in the context of appropriate surgical experience and management of risk factors, including high recipient body mass index, uncontrolled hypertension, low bladder capacity, and vascular calcifications [1]. The role of surgical training and experience is particularly key, given that en bloc kidney acceptance is typically a surgical decision, and our data show that en bloc kidney usage is focused on a small number of transplant centers. Frequent monitoring for thrombosis and appropriate use of anticoagulation must also be considered [6, 31].

## Author Contributions

Engen, Harshman, Lindner, Kumar, Bartosh, and Smith conceptualized and designed the study. Engen and Perkins were involved with the analysis and interpretation of the data. Engen, Harshman, Lindner, Kumar, Puliyanda, Bartosh, and Smith drafted the article. Perkins provided statistical expertise.

## Funding

The authors have nothing to report.

## Supporting information


**Table S1:** En bloc kidney transplants in pediatric recipients by year, 2000–2023, overall and subdivided by kidney‐alone versus multiorgan transplants.
**Table S2:** Number of en bloc kidney transplants as a percentage of all pediatric deceased donor kidney transplants, by center, before and after the implementation of the Kidney Allocation System.
**Table S3:** Demographic and clinical characteristics of pediatric recipients of an en bloc kidney transplant 2000–2024, overall and by transplant type. CAKUT: Congenital Anomaly of the Kidney and Urinary Tract. GN/FSGS: Glomerulonephritis/Focal Segmental Glomerulosclerosis. cPRA: Calculated Panel Reactive Antibody.
**Table S4:** Demographic and clinical characteristics of en bloc kidney donors for pediatric recipients 2000–2024, overall and by transplant type. KDPI: Kidney Donor Profile Index. DCD: Deceased after cardiac death. HLA: Human Leukocyte Antigen.

## Data Availability

This manuscript utilized an analyzable data registry that is publicly available as the Scientific Registry of Transplant Recipients Standard Analysis File. Information on the data file and a request for access are available on the SRTR website: https://www.srtr.org/requesting‐srtr‐data/data‐requests/.
