# Supplementary material for: Use of En Bloc Kidney Allografts for Pediatric Kidney‐Alone and Multiorgan Transplant Recipients in the United States
Source: Pediatr Transplant. 2026 May 13;30:e70330. doi: 10.1111/petr.70330 (PMC13169177; doi:10.1111/petr.70330)
Supplement: Supplementary file 1 — Table S1: En bloc kidney transplants in pediatric recipients by year, 2000–2023, overall and subdivided by kidney‐alone versus multiorgan transplants. Table S2: Number of en bloc kidney transplants as a percentage of all pediatric deceased donor kidney transplants, by center, before and after the implementation of the Kidney Allocation System. Table S3: Demographic and clinical characteristics of pediatric recipients of an en bloc kidney transplant 2000–2024, overall and by transplant type. CAKUT: Congenital Anomaly of the Kidney and Urinary Tract. GN/FSGS: Glomerulonephritis/Focal Segmental Glomerulosclerosis. cPRA: Calculated Panel Reactive Antibody. Table S4: Demographic and clinical characteristics of en bloc kidney donors for pediatric recipients 2000–2024, overall and by transplant type. KDPI: Kidney Donor Profile Index. DCD: Deceased after cardiac death. HLA: Human Leukocyte Antigen. [file PETR-30-e70330-s001.docx]

|  | **All En Bloc** |  | **En Bloc Kidney-alone** | | **En Bloc Multi-organ** | |
| --- | --- | --- | --- | --- | --- | --- |
| **Year** | N |  | n | % | n | % |
| 2000 | 3 |  | 2 | 66.7 | 1 | 33.3 |
| 2001 | 5 |  | 3 | 60 | 2 | 40 |
| 2002 | 11 |  | 8 | 72.7 | 3 | 27.3 |
| 2003 | 4 |  | 3 | 75 | 1 | 25 |
| 2004 | 7 |  | 6 | 85.7 | 1 | 14.3 |
| 2005 | 10 |  | 7 | 70 | 3 | 30 |
| 2006 | 9 |  | 7 | 77.8 | 2 | 22.2 |
| 2007 | 16 |  | 10 | 62.5 | 6 | 37.5 |
| 2008 | 12 |  | 9 | 75 | 3 | 25 |
| 2009 | 12 |  | 5 | 41.7 | 7 | 58.3 |
| 2010 | 11 |  | 6 | 54.5 | 5 | 45.5 |
| 2011 | 11 |  | 6 | 54.5 | 5 | 45.5 |
| 2012 | 16 |  | 9 | 56.3 | 7 | 53.8 |
| 2013 | 5 |  | 3 | 60 | 2 | 40 |
| 2014 | 6 |  | 5 | 83.3 | 1 | 16.7 |
| 2015 | 5 |  | 3 | 60 | 2 | 40 |
| 2016 | 5 |  | 1 | 20 | 4 | 80 |
| 2017 | 2 |  | 1 | 50 | 1 | 50 |
| 2018 | 3 |  | 0 | 0 | 3 | 100 |
| 2019 | 5 |  | 1 | 20 | 4 | 80 |
| 2020 | 3 |  | 0 | 0 | 3 | 100 |
| 2021 | 6 |  | 2 | 33.3 | 4 | 66.7 |
| 2022 | 2 |  | 0 | 0 | 2 | 100 |
| 2023 | 4 |  | 0 | 0 | 4 | 100 |
| 2023 | 3 |  | 0 | 0 | 3 | 100 |

**Supplemental Table 1:** En bloc kidney transplants in pediatric recipients by year, 2000-2023, overall and subdivided by kidney-alone versus multi-organ transplants.

|  |  | 2000-2014 |  |  |  | 2015-2024 |  |  |  |
| --- | --- | --- | --- | --- | --- | --- | --- | --- | --- |
| **Program** | **Deceased Transplants** | **En-Bloc** | **Single** | **Percent En-Bloc** |  | **En_Bloc** | **Single** | **Percent En-Bloc** | **Change in En-Bloc percentage** |
| A | 46 | 1 | 23 | 4.2% |  | 0 | 22 | 0.0% | -100.0% |
| AA | 28 | 1 | 27 | 3.6% |  | 0 | 0 | 0.0% | -100.0% |
| B | 181 | 3 | 101 | 2.9% |  | 1 | 76 | 1.3% | -55.0% |
| BB | 287 | 2 | 190 | 1.0% |  | 1 | 94 | 1.1% | 1.1% |
| C | 31 | 1 | 14 | 6.7% |  | 0 | 16 | 0.0% | -100.0% |
| CC | 54 | 2 | 15 | 11.8% |  | 0 | 37 | 0.0% | -100.0% |
| D | 88 | 3 | 40 | 7.0% |  | 0 | 45 | 0.0% | -100.0% |
| DD | 58 | 5 | 29 | 14.7% |  | 0 | 24 | 0.0% | -100.0% |
| E | 57 | 1 | 36 | 2.7% |  | 0 | 20 | 0.0% | -100.0% |
| EE | 73 | 5 | 33 | 13.2% |  | 0 | 35 | 0.0% | -100.0% |
| F | 96 | 1 | 47 | 2.1% |  | 0 | 48 | 0.0% | -100.0% |
| FF | 13 | 1 | 7 | 12.5% |  | 1 | 4 | 20.0% | 60.0% |
| G | 41 | 0 | 20 | 0.0% |  | 1 | 20 | 4.8% |  |
| GG | 193 | 0 | 73 | 0.0% |  | 8 | 112 | 6.7% |  |
| H | 211 | 2 | 126 | 1.6% |  | 1 | 82 | 1.2% | -22.9% |
| HH | 78 | 2 | 44 | 4.3% |  | 0 | 32 | 0.0% | -100.0% |
| I | 5 | 1 | 3 | 25.0% |  | 0 | 1 | 0.0% | -100.0% |
| II | 48 | 1 | 45 | 2.2% |  | 0 | 2 | 0.0% | -100.0% |
| J | 69 | 2 | 39 | 4.9% |  | 0 | 28 | 0.0% | -100.0% |
| JJ | 261 | 0 | 124 | 0.0% |  | 1 | 136 | 0.7% |  |
| K | 62 | 1 | 20 | 4.8% |  | 0 | 41 | 0.0% | -100.0% |
| KK | 173 | 2 | 110 | 1.8% |  | 1 | 60 | 1.6% | -8.2% |
| L | 147 | 8 | 81 | 9.0% |  | 0 | 58 | 0.0% | -100.0% |
| LL | 98 | 8 | 40 | 16.7% |  | 0 | 50 | 0.0% | -100.0% |
| M | 97 | 4 | 55 | 6.8% |  | 0 | 38 | 0.0% | -100.0% |
| MM | 54 | 2 | 32 | 5.9% |  | 0 | 20 | 0.0% | -100.0% |
| N | 170 | 0 | 109 | 0.0% |  | 4 | 57 | 6.6% |  |
| NN | 128 | 2 | 70 | 2.8% |  | 0 | 56 | 0.0% | -100.0% |
| O | 115 | 4 | 50 | 7.4% |  | 0 | 61 | 0.0% | -100.0% |
| OO | 269 | 5 | 177 | 2.7% |  | 1 | 86 | 1.1% | -58.2% |
| P | 169 | 4 | 94 | 4.1% |  | 2 | 69 | 2.8% | -31.0% |
| PP | 261 | 39 | 108 | 26.5% |  | 9 | 105 | 7.9% | -70.2% |
| Q | 193 | 1 | 95 | 1.0% |  | 0 | 97 | 0.0% | -100.0% |
| QQ | 18 | 1 | 10 | 9.1% |  | 0 | 7 | 0.0% | -100.0% |
| R | 150 | 1 | 71 | 1.4% |  | 0 | 78 | 0.0% | -100.0% |
| S | 44 | 1 | 22 | 4.3% |  | 0 | 21 | 0.0% | -100.0% |
| T | 43 | 2 | 21 | 8.7% |  | 1 | 19 | 5.0% | -42.5% |
| U | 229 | 2 | 115 | 1.7% |  | 1 | 111 | 0.9% | -47.8% |
| V | 57 | 1 | 41 | 2.4% |  | 0 | 15 | 0.0% | -100.0% |
| W | 28 | 1 | 16 | 5.9% |  | 0 | 11 | 0.0% | -100.0% |
| X | 79 | 13 | 28 | 31.7% |  | 5 | 33 | 13.2% | -58.5% |
| Y | 52 | 1 | 32 | 3.0% |  | 0 | 19 | 0.0% | -100.0% |
| Z | 110 | 1 | 57 | 1.7% |  | 0 | 52 | 0.0% | -100.0% |

**Supplemental Table 2:** Number of en bloc kidney transplants as a percentage of all pediatric deceased donor kidney transplants, by center, before and after the implementation of the Kidney Allocation System.

|  |  | All En Bloc  (N=176) | |  | En Bloc Kidney-alone  (N=97) | | En Bloc MOT  (N=79) | |  |
| --- | --- | --- | --- | --- | --- | --- | --- | --- | --- |
|  |  | N/med | %/(IQR) |  | n/med | %/(IQR) | n/med | %/(IQR) | p |
| Age , years |  | 8.5 | (3-18) |  | 13 | (7.5-16) | 4 | (2-8) | <0.001 |
| Male |  | 94 | 53.4% |  | 55 | 56.7% | 39 | 49.4% | 0.36 |
| Height, cm |  | 117 | (85.2-146.9) |  | 145 | (116.9-155) | 92 | (78.7-111.8) | <0.001 |
| Weight, kg |  | 23.8 | (13.6-42) |  | 40.2 | (22.2-51.6) | 15 | (11.7-23) | <0.001 |
| Blood type |  |  |  |  |  |  |  |  | 0.42 |
| A |  | 56 | 31.8% |  | 26 | 26.8% | 30 | 38% |  |
| AB |  | 5 | 2.8% |  | 3 | 3.1% | 2 | 2.5% |  |
| B |  | 20 | 11.4% |  | 13 | 13.4% | 7 | 8.9% |  |
| O |  | 95 | 54% |  | 55 | 56.7% | 40 | 50.6% |  |
| Race/Ethnicity |  |  |  |  |  |  |  |  | 0.57 |
| Asian |  | 6 | 3.4% |  | 4 | 4.1% | 2 | 2.5% |  |
| Black |  | 33 | 18.8% |  | 22 | 22.7% | 11 | 13.9% |  |
| Hispanic |  | 45 | 25.6% |  | 24 | 24.7% | 21 | 26.6% |  |
| Other |  | 7 | 4% |  | 4 | 4.1% | 3 | 3.8% |  |
| White |  | 85 | 48.3% |  | 46 | 44.3% | 42 | 53.2% |  |
| Diagnosis |  |  |  |  |  |  |  |  | <0.001 |
| CAKUT |  | 27 | 15.3% |  | 22 | 22.7% | 5 | 6.3% |  |
| Genetic |  | 28 | 15.9% |  | 10 | 10.3% | 18 | 22.8% |  |
| GN/FSGS |  | 23 | 13.1% |  | 20 | 20.6% | 3 | 3.8% |  |
| Other |  | 80 | 45.5% |  | 34 | 35.1% | 46 | 58.2% |  |
| Re-transplant |  | 18 | 10.2% |  | 11 | 11.3% | 7 | 8.9% |  |
| Dialysis |  |  |  |  |  |  |  |  | <0.001 |
| Preemptive |  | 62 | 35.2% |  | 17 | 17.5% | 45 | 57% |  |
| ≤1 year |  | 50 | 28.4% |  | 34 | 35.1% | 16 | 20.3% |  |
| >1 year-≤3 years |  | 46 | 26.2% |  | 37 | 38.1% | 9 | 11.4% |  |
| >3 years-≤5 years |  | 12 | 6.8% |  | 6 | 6.2% | 6 | 7.6% |  |
| ≥5 years |  | 6 | 3.4% |  | 3 | 3.1% | 3 | 3.8% |  |
| cPRA |  | 0 | (0-0) |  | 0 | (0-1) | 0 | (0-0) | 0.08 |
| missing |  |  |  |  |  |  |  |  |  |

**Supplemental Table 3:**  Demographic and clinical characteristics of pediatric recipients of an en bloc kidney transplant 2000-2024, overall and by transplant type. CAKUT: Congenital Anomaly of the Kidney and Urinary Tract. GN/FSGS: Glomerulonephritis/Focal Segmental Glomerulosclerosis. cPRA: Calculated Panel Reactive Antibody

|  |  | All En Bloc  (N=176) | |  | En Bloc Kidney-alone  (N=97) | | En Bloc MOT  (N=79) | |  |
| --- | --- | --- | --- | --- | --- | --- | --- | --- | --- |
|  |  | N/med | %/(IQR) |  | n/med | %/(IQR) | n/med | %/(IQR) | p |
| Age , years |  | 1 | (1-3) |  | 1 | (0.5-3) | 1 | (1-3) | 0.59 |
| Height, cm |  | 86.4 | (73.7-98) |  | 86 | (72.5-96.3) | 88 | (74-99.1) | 0.38 |
| Weight, kg |  | 12.2 | (9.5-15) |  | 12 | (9.8-15) | 12.7 | (9.3-17.8) | 0.29 |
| Donor-Recipient height ratio |  | 0.76 | (0.6-0.96) |  | 0.64 | (0.54-0.79) | 0.9 | (0.79-1.06) | <0.001 |
| Donor-Recipient weight ratio |  | 0.53 | (0.28-0.84) |  | 0.29 | (0.22-0.53) | 0.74 | (0.57-1.06) | <0.001 |
| Donor-Recipient BSA index |  |  |  |  |  |  |  |  | <0.001 |
| 0≤0.65 |  | 90 | 51.1% |  | 74 | 76.3% | 16 | 20.3% |  |
| >0.65≤1 |  | 54 | 30.7% |  | 15 | 15.5% | 39 | 49.4% |  |
| >1≤1.5 |  | 25 | 14.2% |  | 7 | 7.2% | 18 | 22.8% |  |
| >1.5 |  | 7 | 4% |  | 1 | 1% | 6 | 7.6% |  |
| ABO incompatible |  | 1 | 0.6% |  | 0 |  | 1 | 1.3% | 0.36 |
| KDPI (%) |  | 48 | (42-56) |  | 49 | (43-58) | 46 | (40-54) | 0.03 |
| DCD donor |  | 1 | 0.6% |  | 1 | 1% | 0 |  | 1 |
| HLA mismatch |  |  |  |  |  |  |  |  | 0.07 |
| 0 |  | 4 | 2.3% |  | 4 | 4.1% | 0 |  |  |
| 1-2 |  | 7 | 4% |  | 4 | 4.1% | 3 | 3.8% |  |
| 3 |  | 19 | 10.8% |  | 11 | 11.3% | 8 | 10.1% |  |
| 4 |  | 44 | 25% |  | 28 | 28.9% | 16 | 20.3% |  |
| 5 |  | 74 | 42% |  | 40 | 41.2% | 34 | 43% |  |
| 6 |  | 28 | 15.9% |  | 10 | 10.3% | 18 | 22.8% |  |

**Supplemental Table 4:** Demographic and clinical characteristics of en bloc kidney donors for pediatric recipients 2000-2024, overall and by transplant type. KDPI: Kidney Donor Profile Index. DCD: Deceased after cardiac death. HLA: Human Leukocyte Antigen
